# Association of Direct Oral Anticoagulants (DOACs) and Warfarin With Haemorrhagic Risk by Applying Correspondence Analysis to Data From the Italian Pharmacovigilance Database – A Case Study

**DOI:** 10.3389/fphar.2021.790740

**Published:** 2021-12-07

**Authors:** Mario Gaio, Carmen Ferrajolo, Alessia Zinzi, Consiglia Riccardi, Pasquale Di Filippo, Ludovica Carangelo, Gorizio Pieretti, Francesco Rossi, Giovanni Francesco Nicoletti, Annalisa Capuano

**Affiliations:** ^1^ Campania Regional Centre for Pharmacovigilance and Pharmacoepidemiology, University of Campania “Luigi Vanvitelli”, Department of Experimental Medicine, Napoli, Italy; ^2^ Plastic Surgery Unit, University of Campania “Luigi Vanvitelli”, Multidisciplinary Department of Medical Surgical and Dental Sciences, Napoli, Italy

**Keywords:** pharmacovigilance, spontaneous reporting system, adverse event, drug safety, correspondence analysis, anticoagulants

## Abstract

**Introduction:** Post-marketing data on the risks associated with direct oral anticoagulants (DOACs) are conflicting and only few studies evaluated a comparison between each different DOAC. Real-world data from pharmacovigilance databases can help to better define the safety profile of each DOAC and warfarin. However, Correspondence Analysis (CA) could represent a useful tool in this context.

**Objective:** In the attempt to assess the usefulness of CA as a signal detection pharmacovigilance tool, we applied this method to the Italian Pharmacovigilance Database (RNF, *Rete Nazionale di Farmacovigilanza*), by comparing with disproportionality analysis on warfarin and DOACs.

**Methods:** Study based on AEs sent to RNF by Campania Region from 2008 to 2021, in which warfarin, dabigatran, apixaban, edoxaban or rivaroxaban were reported as suspected drug. AEs were clustered into three Standardized MedDRA Queries (SMQs): Central Nervous System Haemorrhages and Conditions (CNSH), GastroIntestinal Perforation, Ulceration, Obstruction or Haemorrhages (GIPUOH) and other Haemorrhages (HH). Non-haemorrhagic AEs were included in a fourth cluster (nHH).

**Results:** We retrieved 1,161 reports: 41.5% are associated to warfarin, 21.0% to dabigatran, 17.8% to rivaroxaban, 13.9% to apixaban and 5.8% to edoxaban. No significant differences in age distribution were observed. Results of CA showed that dabigatran and warfarin have the highest contribution (44.910 and 47.656, respectively) to the inertia of Dimension 1 as well as apixaban and dabigatran to the inertia of Dimension 2 (53.768 and 30.488, respectively). Edoxaban and rivaroxaban showed a negligible total contribution. CA biplot showed positive associations between warfarin and HH, apixaban and CNSH and dabigatran and nHH.

**Conclusion:** Results seem to confirm that DOACs are not interchangeable. Apixaban was surprisingly associated with a higher risk of cerebral haemorrhage. As expected, our data support the better safety profile of DOACs than warfarin in terms of skin and respiratory tract hemorrhagic risks. Finally, we showed how CA could play a complementary role in analyzing data from pharmacovigilance databases.

## Introduction

The oral anticoagulant vitamin K antagonist (VKA) drugs such as warfarin have been widely used for decades as the only oral anticoagulant. Despite its known effectiveness, a frequent dose adjustment is required to maintain its therapeutic range, and risk of haemorrhage is significant. The introduction of direct oral anticoagulants (DOACs)—apixaban, dabigatran, edoxaban, and rivaroxaban—allowed clinicians to overcome these limits. In fact, DOACs have shown a lower risk of haemorrhage than that for warfarin; moreover, the unsolicited routine monitoring with the use of DOACs is much more convenient for patients. In the pivotal randomized clinical trials (RCTs), each DOAC was compared to warfarin, but no head-to-head comparison between the individual DOAC has been performed (Connolly et al., 2009; Granger et al., 2011; Patel et al., 2011; Giugliano et al., 2013). Some unanswered safety aspects remain to address, and observational studies utilizing real-world data could play a complementary role to RCTs. Several observational studies have currently emerged to provide supportive evidence of the safety and/or effectiveness of DOACs in a real-world setting (Keshishian et al., 2016; Kohsaka et al., 2017; Li et al., 2017; Kohsaka et al., 2018; Russo-Alvarez et al., 2018; Coleman et al., 2019; Ikeda et al., 2019; Inoue et al., 2019). Although the increasingly widespread use of DOACs, few studies evaluated their safety profile by leveraging pharmacovigilance data. Specifically, results from a study based on reports of suspected adverse drug reactions held in VigiBase have shown, as well as premarketing authorization RCTs, a reduced risk of intracranial haemorrhage, but an increased risk of gastrointestinal haemorrhage in patients treated with DOACs compared to warfarin (Monaco et al., 2017). Moreover, this study has shown several differences between different DOACs in the rate and type of suspected adverse events (AEs). Another study based on spontaneous reporting data from the Japanese database of Pharmaceuticals and Medical Devices Agency (PMDA) has shown that differences in drug safety aspects may exist between dabigatran and FXa inhibitors (apixaban, edoxaban and rivaroxaban), especially in terms of hemorrhagic and ischemic cerebrovascular events (Terayama, 2017). As already demonstrated, “Correspondence Analysis” (CA) could be a useful tool to uncover the relationships among categorical variables (Sourial et al., 2010). In fact, CA has become a popular method for the analysis of data in a wide range of fields, such as archeology, paleontology, linguistics, marketing, social sciences and more (Freudenthal et al., 2009; Alberti, 2013; Glynn, 2014; Hoare and Bock, 2019). However, CA is rarely use in health sciences. Its first use in health sciences was proposed by Greenacre: he introduced CA in an initial simple example using data on the relationship between headache types and age; then, he illustrated a more complex situation when several categorical variables are involved using test data on a collection of bacterial isolates, with the aim of comparing bacterial types and understanding the inter-relationships of the different tests (Greenacre, 1992).

CA is a multivariate graphical technique which contains three basic concepts: 1) that of a point in a multidimensional space; 2) a weight (or “mass”) assigned to each point; 3) a distance function between the points, called the chi-square distance. Once these three concepts are defined, the aim of CA is to reduce the dimensionality of the points by projecting them onto a subspace, a two-dimensional plane (CA “biplot”) that can be seen as a spatial map of the data. In this subspace, each point is weighted by its respective mass, and the measurement of distance between points and subspace is in terms of chi-square distance. In its simplest form, CA applies to a two-way cross-tabulation (Greenacre, 1984; Greenacre, 2007). In pharmacovigilance studies, researchers often interested in exploring the relationships among categorical variables with the goal of examining associations among these variables (Ferrajolo et al., 2014; Donati et al., 2016). One might consider conducting separate chi-square tests (one for each pair of variables), but this pairwise strategy would quickly become cumbersome and render the results difficult to summarize. More importantly, it would not provide us with a global picture of the salient relationships among these variables when taken together. CA approach explores these relationships “simultaneously” (Sourial et al., 2010). Given that, we believe that the use of CA should be considered in pharmacovigilance studies in addition to the established measures (e.g., the Reporting Odds Ratio, ROR), especially if high dimensional categorical data are investigated. As demonstrative example of using CA in this area, this study analyses the association of suspected AEs with the use of DOACs and warfarin, focusing on bleeding events.

## Materials and Methods

### Data Source

Individual Case Safety Reports (ICSRs) with an oral anticoagulant as a suspected drug were retrieved from the Italian National database for Pharmacovigilance (Rete Nazionale di Farmacovigilanza, RNF). The database collects all ICSRs reported spontaneously or deriving from active pharmacovigilance projects or observational studies. We obtained data elements for the following details: patients’ age and gender, AEs and their seriousness, and type of suspected drug. For this study, only ICSRs sent to the RNF by one Italian Region (Campania) from January 1, 2008 to January 1, 2021 were collected.

### Descriptive Analysis

We performed a descriptive analysis by comparing ICSRs in terms of gender and age, reported AEs, and seriousness for each suspected drug. However, given that old age could be a significant risk factor for bleeding complications, we performed a nonparametric test (Kruskal-Wallis test) for comparing the age of the different populations because the data were not normally distributed (Hughes and Lip, 2007).

Reported AEs are coded according to the Medical Dictionary for Regulatory Activities (MedDRA) and analyzed based on Standardized MedDRA Queries (SMQs). SMQs are validated, standard sets of MedDRA terms that have undergone extensive review, testing, analysis, and expert discussion (MedDRA, 2021). Specifically, the low-level terms (LLTs) and the preferred terms (PTs) reported in our dataset were clustered into three SMQs: “Central Nervous System Haemorrhages and Conditions” (denoted “CNSH” in this paper), “GastroIntestinal Perforation, Ulceration, Obstruction or Haemorrhages” (denoted “GIPUOH” in this paper) and “Haemorrhage” (denoted “HH” in this paper) (in particular, this latter SMQ mainly includes skin, urinary and respiratory tract hemorrhages). LLTs and PTs which were not included in any of these SMQs were grouped in a fourth cluster of non-Haemorrhagic AEs (denoted “nHH” in this paper).

### Correspondence Analysis

We graphically illustrated the most important relationships among the variables using Correspondence Analysis (CA) (Benzécri, 1992). CA is a statistical method designed specifically for the analysis of categorical variables, by building the contingency table through the row and column frequencies (“row profiles” and “column profiles,” as they are called in CA). In our study, the frequencies of different SMQs within each suspected drug have been called “row profile” and the frequencies of the different suspected drugs within each SMQ have been called “column profile.” Moreover, the average row profile is defined as the average of the row profiles weighted by the marginal row frequencies; likewise, the average column profile is defined as the average of the column profiles weighted by the marginal column frequencies (Greenacre, 1984). The “inertia” is a crucial concept in CA: it represents a measure of variance or dispersion of the individual profiles around the average profiles; the larger the variance is, the larger inertia will be. Another useful measure is the Pearson’s chi-square statistic, directly related to the inertia. Positive values of these Pearson residuals indicate a strong association between the variables (i.e., stronger than expected under the basic assumption of no relationship) and negative residuals indicate a weak association (i.e., lower than expected under the basic assumption of no relationship). CA decomposes the inertia by identifying a first dimension (Dim. 1) which represents the most important deviations from independence (or the largest amount of explained inertia), a second dimension (Dim. 2) which represents the second largest deviations from independence, and so on (Sourial et al., 2010). Each dimension has an “eigenvalue” that is proportional to the amount of variance explained by each axis. The first axis will include the largest eigenvalue which will be lower in the following axes. We considered the eigenvalues to establish the number of axes to retain. Although CA does not provide any rules on the choice of the number of dimensions for the data analysis, it is usually kept the first few dimensions whose 80% or more variation is explained. Finally, in order to identify and visualize the relationship between the five drugs and the four AE groups, we graphically showed the results of CA in a biplot with row and column profiles as points. The procedure of the biplot analysis should follow two steps: first, the comparison of each category of the same variable on their proximity to the axes of the biplot; second, the comparison of proximity among categories of different variables. Generally, if one or more categories of the same variable are in close proximity on horizontal or vertical axis, it means there are little differences among them on that axis; if the categories of different variables are in close proximity, they are associated with each other. The closer the proximity, the stronger the association. The distance between the categories and the origin of axis measures the quality of the categories on the factor map: categories that are away from the origin are well represented on the factor map (Greenacre, 2017). All statistical analysis was performed using R Statistical Software (version 4.0.3; R Foundation for Statistical Computing, Wien, Austria).

### Disproportionality Analysis

The ROR with a 95% of Confidence Interval (95% CI) was computed to compare the results of CA. This disproportionality analysis aimed to assess if oral anticoagulants have a lower/higher probability of reporting ICSRs with haemorrhagic events compared with warfarin and with each other.

## Results

### Characteristics of ICSRs

**FIGURE 1:**
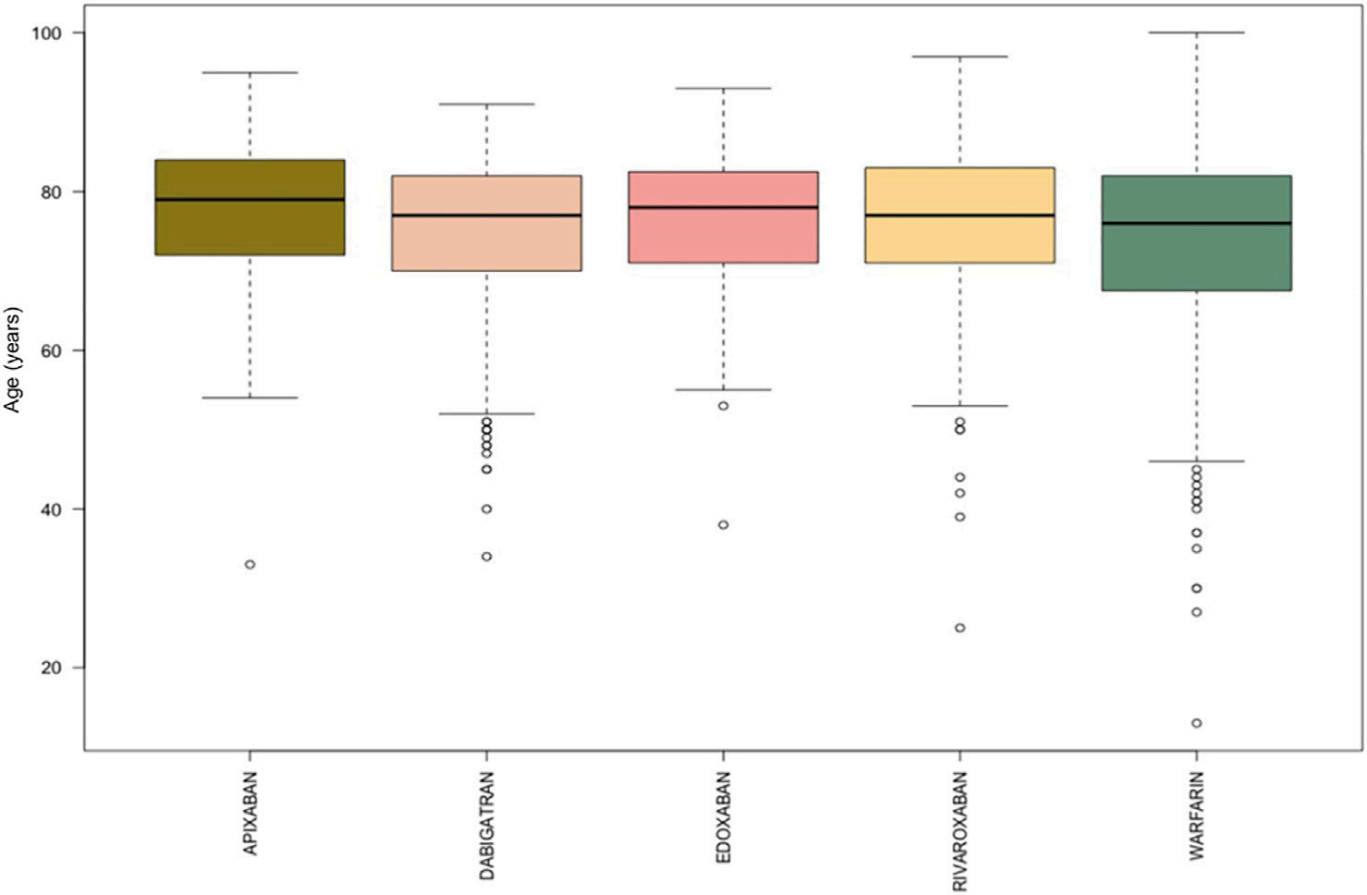
Comparing median age of apixaban, dabigatran, edoxaban, rivaroxaban, and warfarin-related ICSRs reported in Campania Region and collected into the RNF from January 1, 2008, to January 1, 2021.

During the study period, 1,161 ICSRs with apixaban, dabigatran, edoxaban, rivaroxaban or warfarin as suspected drug collected into the RNF were reported in Campania Region. Focusing on these reports, 482 (41.5%) ICSRs were associated to warfarin as suspected drug, followed by dabigatran (*N* = 244, 21.0%), rivaroxaban (*N* = 207, 17.8%), apixaban (*N* = 161, 13.9%) and edoxaban (*N* = 67, 5.8%). Excluding the ICSRs with gender missing information (*N* = 8), ICSRs were equally distributed between genders without relevant differences between each drug. Overall, most of the ICSRs (*N* = 722, 62.2%) was classified as serious, but considerable variability of seriousness was observed between ICSRs stratified by suspected drugs. In particular, more than 70% of the ICSRs associated to apixaban, edoxaban and rivaroxaban were classified as serious (75.2, 79.1, and 71.0%, respectively) in contrast to dabigatran and warfarin-related ICSRs in which serious AEs were less frequently reported (50.8 and 55.8%, respectively). Moreover, the overall average age of patients was 74.8 years (±11.0), and small differences emerged comparing median age of ICSRs for each drug (Table 1, Figure 1).

**TABLE 1:** Oral anticoagulants related ICSRs reported in Campania Region and collected into RNF from January 1, 2008 to January 1, 2021 stratified by gender, age and seriousness.

	Apixaban	Dabigatran	Edoxaban	Rivaroxaban	Warfarin	Tot. ICSRs
Gender (%)						
F	80 (49.7)	127 (52.5)	30 (44.8)	95 (45.9)	243 (50.4)	575 (49.5)
M	80 (49.7)	115 (47.5)	37 (55.2)	110 (53.1)	236 (49.0)	578 (49.8)
NA	1 (0.6)	—	—	2 (1.0)	3 (0.6)	8 (0.7)
Age						
Mean (SD)	77.4 (±8.9)	74.5 (±10.9)	75.8 (±9.8)	75.5 (±10.8)	73.7 (±11.9)	74.8 (±11.0)
Range	33–95	34–91	38–93	25–97	13–100	13–100
Seriousness						
Serious	121 (75.2)	124 (50.8)	53 (79.1)	147 (71.0)	277 (55.8)	722 (61.4)
Not serious	34 (21.1)	118 (48.4)	14 (20.9)	54 (26.1)	181 (36.5)	401 (34.1)
NA	6 (3.7)	2 (0.8)	—	6 (2.9)	38 (7.7)	52 (4.4)
Tot. ICSRs	161 (13.9)	244 (21.0)	64 (5.5)	207 (17.8)	482 (41.5)	1,161 (100.0)

### Patterns of AE’s for Each Drug

**FIGURE 2:**
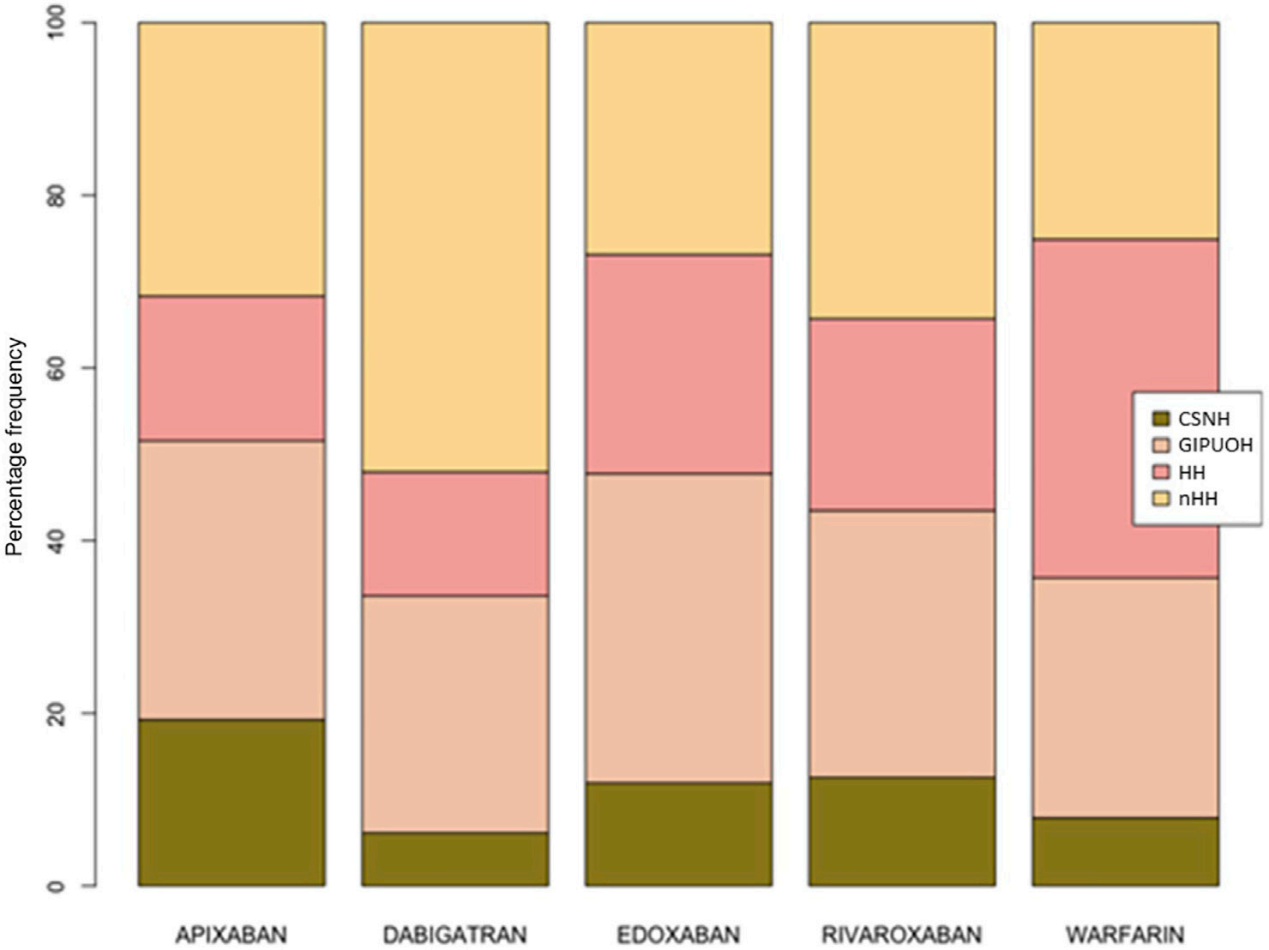
Row profiles of drugs and AEs.

There were different patterns of the frequencies of reported AEs for each DOAC and warfarin (Tables 2, 3, Figure 2). Row profiles correspond to the relative frequencies of the different AEs reported with each drug. For example, among reports with apixaban as suspected drug, GIPUOH is the most reported AE (32.3%) followed by nHH (31.7%), CNSH (19.2%) and HH (16.8%). In our analysis, the average row profile, presented in the bottom row of Table 3, shows that, when pooling across drugs, nHH is the most common AE while CNSH is the least common. Analogously, column profiles are the relative frequencies of the different drugs within each AEs. For example, among reports with GIPUOH as suspected AE, the most common involved drug is warfarin (39.3%), followed by dabigatran (19.7%), rivaroxaban (18.8%), apixaban (15.3%) and edoxaban (7.0%). The average column profile shows that, when pooling across AEs, warfarin is the most common drug while edoxaban is the least common. Given that, we can compare row profiles or column profiles observing the “distance” from their average row profile or average column profile.

**TABLE 2:** Frequency of observed AEs by drug, clustered into four MedDRA SMQs (GIPUOH, GastroIntestinal Perforation, Ulceration, Obstruction or Haemorrhages SMQ; CNSH, Central Nervous System Haemorrhages and Conditions SMQ; HH, Haemorrhages SMQ; nHH, non-Haemorrhagic AEs).

	GIPUOH	CNSH	HH	nHH	Row marginals
Apixaban	52	31	27	51	161
Dabigatran	67	15	35	127	244
Edoxaban	24	8	17	18	67
Rivaroxaban	64	26	46	71	207
Warfarin	134	38	189	121	482
Column marginals	341	118	314	388	1,161

**TABLE 3:** Row and column profiles of drugs and AEs.

Row Profiles (%)							
		Adverse events					
		GIPUOH	CNSH	HH		nHH	Total
Drugs	Apixaban	32.3	19.2	16.8		31.7	100.0
	Dabigatran	27.5	6.1	14.3		52.1	100.0
	Edoxaban	35.8	11.9	25.4		26.9	100.0
	Rivaroxaban	30.9	12.6	22.2		34.3	100.0
	Warfarin	27.8	7.9	39.2		25.1	100.0
Average row profile		29.4	10.2	27.0		33.4	100.0
**Column Profiles (%)**							
		**Adverse events**					
		**GIPUOH**	**CNSH**	**HH**	**nHH**		**Average column profile**
Drugs	Apixaban	15.3	26.3	8.6	13.1		15.8
	Dabigatran	19.7	12.7	11.1	32.8		19.1
	Edoxaban	7.0	6.8	5.4	4.7		6.0
	Rivaroxaban	18.8	22.0	14.7	18.3		18.5
	Warfarin	39.3	32.2	60.2	31.2		40.8
Total		100.0	100.0	100.0	100.0		100.0

### Correspondence Analysis

**FIGURE 3:**
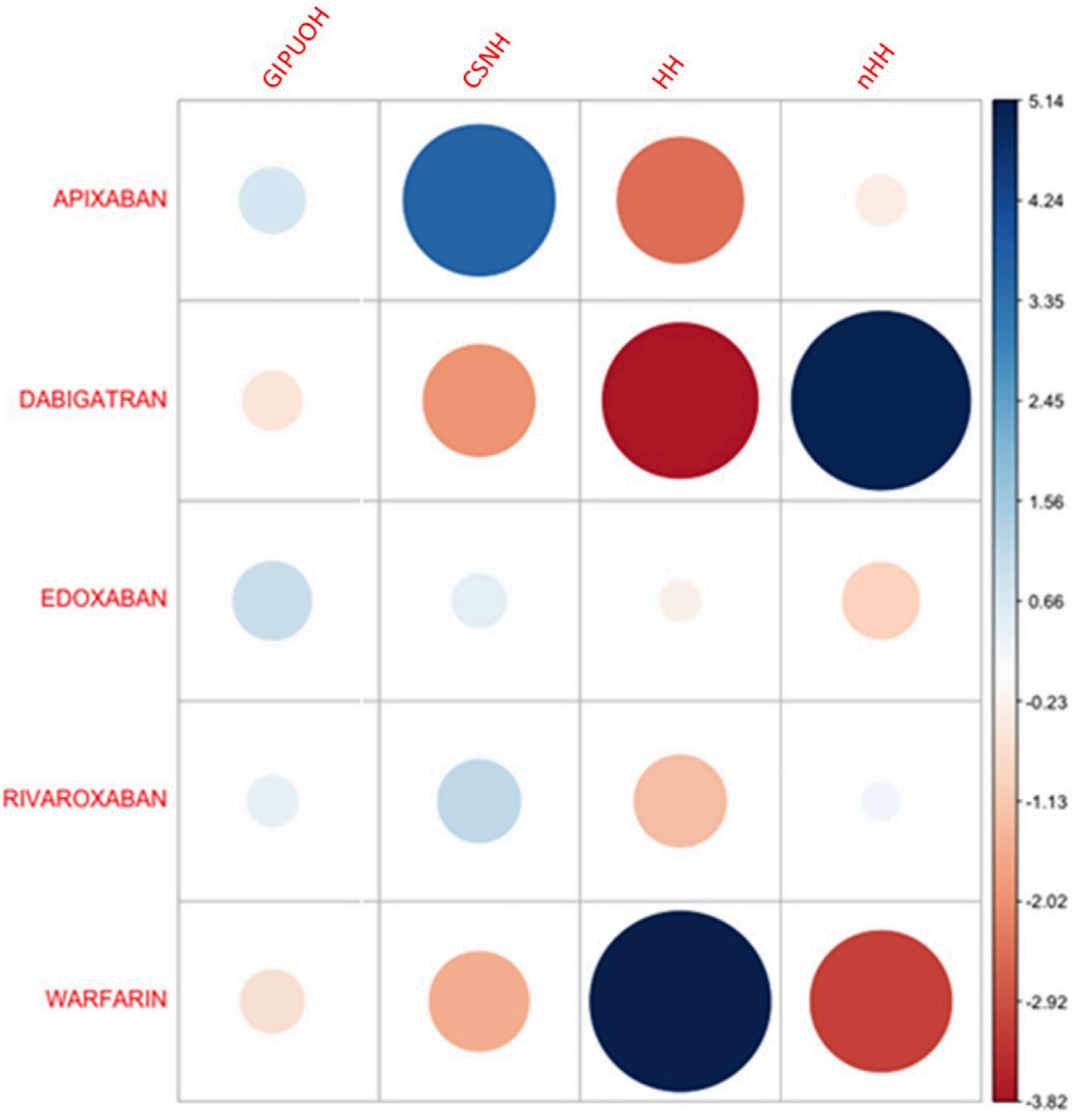
Pearson residuals–Positive residuals are in blue, negative residuals are in red. The size of the circle is proportional to the amount of the cell contribution.

**FIGURE 4:**
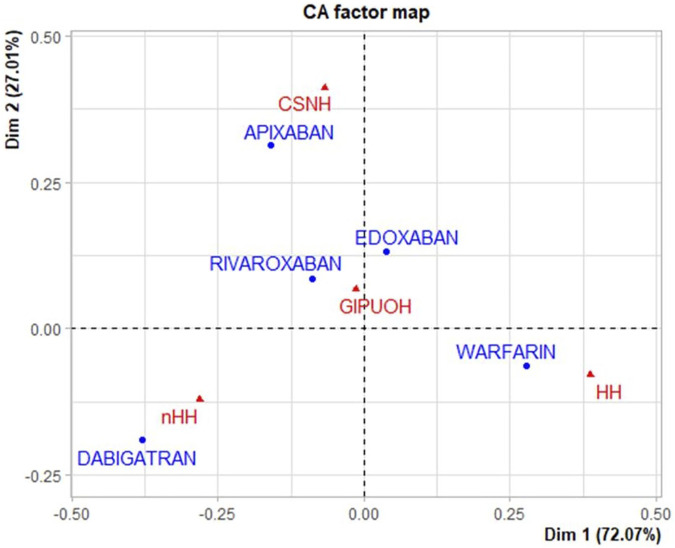
Correspondence analysis biplot of AEs and oral anticoagulants.

The eigenvalues of dimension 1 and dimension 2 were 0.067 and 0.025, respectively, so the first two dimension represented 99.08% of the variance (see Supplementary Table S1). The factor loading matrix of drugs and AEs on dimension 1 and dimension 2 was showed in Supplementary Table S2. It can show how much each drug has contributed to the inertia of each dimension. Dabigatran and warfarin have shown the highest contribution (44.910 and 47.656, respectively) to the inertia of Dimension 1, and apixaban and dabigatran have shown the highest contribution (53.768 and 30.488, respectively) to the inertia of Dimension 2. Edoxaban and rivaroxaban have shown a negligible contribution for each dimension. We obtained the Pearson residuals to understand how each variable contributed the most to the χ^2^ score: higher the standardized residual (in absolute value), higher the contribute to the χ^2^ score (Table 4). To better visualize the results, a balloon plot has been graphed: positive Pearson residuals are shown in blue, negative Pearson residuals are shown in red; the larger the circle, the greater the contribution (Figure 3). It has showed a strong association between warfarin and HH (5.136), dabigatran and nHH (5.034), and apixaban and CNSH (3.618); moreover, results have showed a moderate repulsion (weak association) between warfarin and CNSH (−1.570) and dabigatran and CNSH (−1.968), and a strong repulsion (very weak association) between warfarin and nHH (−3.158), apixaban and HH (−2.507). Since dimension 1 (72.07%) revealed much more information than dimension 2 (27.01%), we can only see the results on dimension 1 (horizontal axis of Figure 4). Observing the dimension 1 of the biplot only (horizontal axis of Figure 4), we could approximately split the drugs in two categories: DOACs (dabigatran, apixaban, rivaroxaban, and edoxaban) and warfarin; doing analogously with the distribution of the AEs in the biplot, we could approximately split the AEs in two categories: the first including nHH, CNSH, GIPUOH and the second including HH. Finally, according to the spatial distribution of variables, warfarin resulted associated with HH, apixaban was associated with CNSH and dabigatran was associated with nHH. The contribution of edoxaban, rivaroxaban and GIPUOH is negligible because of their proximity to the origin of the axis.

**TABLE 4:** Individual contribution to the Pearson $\chi^{2}$ statistic.

	GIPUOH	CNSH	HH	nHH
Apixaban	0.685	3.618	−2.507	−0.382
Dabigatran	−0.551	−1.968	−3.815	5.034
Edoxaban	0.974	0.456	−0.263	−0.928
Rivaroxaban	0.411	1.082	−1.334	0.219
Warfarin	−0.636	−1.570	5.136	−3.158

### Disproportionality Analysis (Reporting Odds Ratio)

In the disproportionality analysis, apixaban had an increased probability of reporting CNSH in comparison to all other oral anticoagulants (ROR 2.50, 95% CI 1.60–3.92, *p* < 0.05). Moreover, warfarin was associated with an increased probability of reporting HH events if compared with all DOACs (ROR 2.85, 95% CI 2.18–3.73, *p* < 0.05), also if individually compared with apixaban, dabigatran, edoxaban and rivaroxaban (ROR 3.20, 95% CI 2.03–5.03, *p* < 0.05; ROR 3.85, 95% CI 2.58–5.76, *p* < 0.05; ROR 1.90, 95% CI 1.06–3.39, *p* < 0.05; ROR 2.25, 95% CI 1.55–3.29, *p* < 0.05, respectively). Moreover, we observed a statistically significant association between dabigatran and ICSRs with nHH events (ROR 2.73, 95% CI 2.04–3.64, *p* < 0.05), also if individually compared with apixaban, edoxaban, rivaroxaban and warfarin (ROR 2.34, 95% CI 1.54–3.55, *p* < 0.05; ROR 2.95, 95% CI 1.63–5.36, *p* < 0.05; ROR 2.08, 95% CI 1.42–3.04, *p* < 0.05; ROR 3.24, 95% CI 2.34–4.48, *p* > 0.05, respectively).

## Discussion

The present study evaluated the safety profile of warfarin and DOACs using real-world data sent to the RNF by the Italian Region Campania. During the study period, we analyzed 1,161 ICSRs differently distributed between drug-populations. In line with their date of approval, the most common reported drug was warfarin, followed by dabigatran, rivaroxaban, apixaban and edoxaban. In fact, while warfarin was first introduced in the second half of the 20th century, the DOAC dabigatran and rivaroxaban were both introduced in 2008, apixaban in 2011, and edoxaban in 2015 (EMA, 2021a; EMA, 2021b; EMA, 2021c; EMA, 2021d). The overall average age of patients was 74.8 years (±11.0) with no significant differences in age distribution. Even if age remains one of the strongest risk factors for stroke in patients with atrial fibrillation (AF), real-world data suggest that a significant proportion of older patients are still not receiving stroke prophylaxis treatment in line with guideline recommendations, even in the absence of contraindications to oral anticoagulants (Steinberg et al., 2015). In addition to that, other important factors could influence the safety issue of the DOACs: the indication of use for which the DOAC is given, its dose, the concomitant drugs, linked to the potential risk of pharmacokinetic and pharmacodynamic drug-drug interaction, the comorbidities, and the duration of therapy (Roberti et al., 2021).

ICSRs related to apixaban, rivaroxaban and edoxaban are prevalently associated to serious AEs. Actually, as described in their Summaries of Product Characteristics, apixaban and edoxaban are used in patients who have one or more risk factors, such as having had a previous stroke, having high blood pressure, diabetes, heart failure or being 75 years old or over (EMA, 2021c; EMA, 2021d).

To our knowledge, this is the first study that analyses real-world safety data using the statistical tool of Correspondence Analysis. It is based on the analysis of the contingency table through the row and column profiles to present a unique graphical display showing the relationship among variables (Zhu et al., 2017). CA could represent an additional tool in exploring the relationship among sets of categorical variables. It is a geometric approach for visualizing the rows and columns of a two-way contingency table as points in a low-dimensional space (a representation which retains some meaningful properties of the original data). The aim of CA is to have a global view of the data that is useful for interpretation, also in terms of chi-square distance between different modalities of the same variables. Therefore, based on constituent ratios, we used CA to reveal the relationship between oral anticoagulants and AEs. Results showed that warfarin is associated to AEs grouped in HH, which mainly includes skin, urinary and respiratory tract hemorrhages. Apixaban was associated with CNSH, and dabigatran was associated with nHH. Moreover, dabigatran and apixaban showed a non-association with HH, and warfarin showed a non-association with nHH. Our results cannot confirm the increased tendency of DOACs to develop gastrointestinal bleeding compared to warfarin. Compared to warfarin, DOACS’ lower bioavailability (dabigatran 7%, rivaroxaban 66%, apixaban 50%, edoxaban 68%) suggests a potentially higher persistence in the gastrointestinal lumen with the resulting increased risk of bleeding particularly in case of high dosage (Desai et al., 2013). Our findings are conflicting with those of pre-marketing authorization RCTs, which showed an increased risk of gastrointestinal haemorrhage of DOACs against warfarin (Loffredo et al., 2015). Specifically, as described in the meta-analysis by Loffredo et al., rivaroxaban and high dosages of edoxaban and dabigatran significantly increased gastrointestinal bleeding against warfarin while a null effect was detected with apixaban. Conversely, some register-based observational studies identified no differences in the rates of gastrointestinal bleeding (Abraham et al., 2015; Chang et al., 2015; Sjögren et al., 2017). From our data, even if CA biplot showed a proximity of GIPUOH with edoxaban and rivaroxaban, we cannot conclude that edoxaban and rivaroxaban are associated with GIPUOH, because of their low contribution to both dimensions.

Relating to intracranial haemorrhages, our findings are contrary to the current evidence. In fact, according to the spatial distribution in CA biplot, apixaban resulted associated with CNSH while results from pivotal study showed a significant reduction of risk of bleeding with apixaban against warfarin (Lopes et al., 2010; Granger et al., 2011). The nationwide cohort study by Staerk et al. also showed that treatment with apixaban, as well as dabigatran, was associated with a significant lower risk of intracranial bleeding, compared with warfarin (Staerk et al., 2017). Our finding could be noteworthy, even though it could reflect limitations in pharmacovigilance databases. As expected, our results described a weak association between dabigatran and CSNH, but warfarin showed a similar result.

In line with RCTs and its summary of product characteristics, warfarin showed a high association to HH, which includes respiratory tract haemorrhage such as epistaxis and hemoptysis, genitourinary tract haemorrhage such as hematuria and menorrhagia, and skin bleeding such as ecchymosis and petechiae. Moreover, the association between dabigatran and nHH could suggest its overall safety in term of haemorrhagic events.

Disproportionality analysis with ROR confirmed the results of CA, that could suggest that CA is a reliable method. Moreover, in this analysis, we believe that CA showed many advantages over ROR in terms of summarizing and visualizing data and results. Unlike the pairwise comparison of ROR (i.e., apixaban/CNSH vs. dabigatran/CNSH), CA explores relationships between each category of each variable simultaneously; it could be useful in a context where there are many categories (and many variables, in the case of “Multiple CA”). Moreover, in contrast to the conventional statistical methods such as ROR, CA is not a confirmatory technique, trying to prove a hypothesis, but rather an exploratory technique, trying to reveal the data content. As described by Greenacre, CA could serve as a window onto the data, allowing medical researchers easier access to their results and facilitating discussion of the data and possibly generating hypotheses which can be formally tested at a later stage (Greenacre, 1992).

## Conclusion

Both strengths and limitations are related to the study data source, a large database of spontaneously reporting adverse drug events. This kind of studies reflects both real-life events and, partially, real-life drug use, including drug use patterns that cannot be studied in clinical trials for ethical reasons (Rafaniello et al., 2020). However, several limitations need to be considered while interpreting this type of results. On the top of that, under-reporting phenomena (i.e., only a minority of AEs might be identified and reported) is highly probably in a spontaneous report system, leading to an underestimation of the real frequency of adverse events. Moreover, this type of data does not include information on drug-exposed population, thus we cannot estimate the incidence of these events among treated patients. Finally, as already discussed, our analysis lacks an assessment of the main confounders which could explain the differences of DOACs in terms of safety profile. Given that, our analysis has not been intended to detect new safety signal, but to assess the value of CA in pharmacovigilance studies. We showed that CA could represent an alternative or complementary methodological approach to the disproportionality analysis in assessing data from pharmacovigilance databases.

## Data Availability

The raw data supporting the conclusion of this article will be made available by the authors, without undue reservation.
