# Supplementary material for: Association of Direct Oral Anticoagulants (DOACs) and Warfarin With Haemorrhagic Risk by Applying Correspondence Analysis to Data From the Italian Pharmacovigilance Database – A Case Study
Source: Front Pharmacol. 2021 Dec 7;12:790740. doi: 10.3389/fphar.2021.790740 (PMC8691542; doi:10.3389/fphar.2021.790740)
Supplement: Supplementary file 1 [file DataSheet1.docx]

Supplementary Material

**Supplementary Table 1** Statistics summary of correspondence analysis – eigenvalues

|  | **Dim. 1** | **Dim. 2** | **Dim. 3** |
| --- | --- | --- | --- |
| Variance | 0.067 | 0.025 | 0.001 |
| % of variance | 72.072 | 27.008 | 0.920 |
| Cumulative % of var. | 72.072 | 99.080 | 100.000 |

**Supplementary Table 2** Factor loading matrix of correspondence analysis

| **Variable** | **Dim. 1** | **Contribution** | **Dim. 2** | **Contribution** |
| --- | --- | --- | --- | --- |
| *Drugs* |  |  |  |  |
| Apixaban | -0.160 | 5.239 | 0.313 | 53.768 |
| Dabigatran | -0.379 | 44.910 | -0.191 | 30.488 |
| Edoxaban | 0.038 | 0.123 | 0.132 | 3.988 |
| Rivaroxaban | -0.088 | 2.072 | 0.086 | 5.236 |
| Warfarin | 0.278 | 47.656 | -0.063 | 6.520 |
| *Adverse drug reactions* |  |  |  |  |
| Gastrointestinal haemorrhages | -0.014 | 0.081 | 0.068 | 5.396 |
| CNS haemorrhages | -0.067 | 0.687 | 0.412 | 68.441 |
| Other haemorrhages | 0.387 | 60.137 | 0.079 | 6.698 |
| Non-haemorrhagic AEs | 0.281 | 39.095 | -0.121 | 19.465 |
